# EHMTI-0163. Reduced neck and shoulder strength in patients with tension-type headache. A case control study

**DOI:** 10.1186/1129-2377-15-S1-C40

**Published:** 2014-09-18

**Authors:** BK Madsen, K Søgaard, LL Andersen, JH Skotte, RH Jensen

**Affiliations:** 1 Neurology, Danish Headache Center, Glostrup, Denmark; 2 Physical Activity and Health in Work Life, Institute of Sports Science and Clinical Biomechanics University of Southern Denmark, Odense, Denmark; 3 NRCWE, National Research Centre for the Working Environment, Copenhagen, Denmark

## Background

Tension-type headache (TTH) is associated with increased muscle tenderness, with an increasing headache frequency and intensity. The potential role of the peripheral muscles in TTH is unclear, and it is unknown if tenderness is related to strength.

## Aims

To compare muscle strength in neck and shoulder in TTH patients and healthy controls, by examining the Maximal Voluntary Isometric Contraction (MVC) during shoulder abduction, neck flexion and extension as well as the extension-flexion strength ratio of the neck.

## Methods

60 TTH patients and, 30 sex and aged matched healthy controls were included. Inclusion criteria for patients TTH ≥8 days per month. The MVC in neck extensor and flexor muscles were tested with the subject seated upright. MVC in shoulder abduction was tested with the subject lying supine. The MVC tests were performed using a computerized dynamometer.

## Results

Compared to controls TTH patients had a significantly weaker neck extension (21.49±10.31Nm) vs. (17.07±9.16 Nm) (p= 0.02) resulting in a significantly lower Extension/Flexion moment ratio (p=0.03). TTH-patients also showed a tendency to significantly lower shoulder abduction strength (44.3±19.3 Nm) vs. (38.7, ±15.9 Nm.) (p = 0.05).

## Conclusions

The reduced neck Extension/Flexion ratio due to decreased strength of the neck extensors and the borderline lower shoulder abduction strength, suggests an unbalanced muscle activity in TTH patients.

No conflict of interest.

